# Rapid, portable Epstein‒Barr virus DNA detection using enzymatic recombinase amplification combined with the CRISPR–Cas12a system

**DOI:** 10.1002/ctm2.70028

**Published:** 2024-09-23

**Authors:** Jia Li, Hao Cheng, Xiaojun Wang, Ning Chen, Liujie Chen, Lili Duan, Fenghua Tan, Kai Li, Duanfang Liao, Zheng Hu

**Affiliations:** ^1^ The First Clinical College of Xiangnan University the First Affiliated Hospital of Xiangnan University the First People's Hospital of Chenzhou Chenzhou China; ^2^ Translational Medicine Institute the First People's Hospital of Chenzhou Hengyang Medical School University of South China Chenzhou China; ^3^ Department of Nasopharyngeal Carcinoma the First People's Hospital of Chenzhou Hengyang Medical School University of South China Chenzhou China; ^4^ Department of Cancer Center the Second Affiliated Hospital Hengyang Medical School University of South China Hengyang China; ^5^ The Oncology Department of the First People's Hospital of Chenzhou Chenzhou China; ^6^ National Engineering Research Center of Personalized Diagnostic and Therapeutic Technology Hunan University of Chinese Medicine Changsha China

Dear Editor,

Nasopharyngeal carcinoma (NPC), a malignancy affecting the head and neck region, is prevalent in the southern and southeastern coastal regions of China. The primary cause of NPC is the Epstein−Barr virus (EBV).^1^ EBV DNA detection is crucial for the screening and monitoring of NPC and other EBV infection‐related diseases. Plasma EBV DNA is considered an important indicator for early NPC screening,^2^ as well as monitoring NPC prognosis and treatment efficacy.^3^ However, the clinical diagnostic method involves quantitative polymerase chain reaction (qPCR), the application of which is limited by its time, cost and convenience.^4^ Recently, rapid detection techniques that combine the CRISPR‐Cas system with isothermal amplification technique (for instance recombinase polymerase amplification [RPA], rolling circle amplification [RCA] and loop‐mediated isothermal amplification [LAMP]) have been increasingly developed and used for identifying various pathogens, (e.g. SARS‐CoV‐2,^5^ HPV16/18,^6^ HIV^7^). Enzymatic recombination amplification (ERA) is an advanced version of isothermal amplification technology,^8^ building on RPA technology. Given its efficiency, adaptability and robustness, ERA is a promising method for enhancing the sensitivity of CRISPR‐based pathogen detection.^9^ In this study, we developed a rapid, portable method for detecting EBV nucleic acids by ERA combined with CRISPR–Cas12a (ERA–Cas12a).

Firstly, we tested the enhanced effect of ERA amplification to CRISPR–Cas12a detection of EB DNA by CRISPR–Cas12a‐mediated fluorescence cleavage assay. EBV DNA samples that were not pre‐amplified by ERA showed no notable alteration of fluorescence intensity contrast to the negative control (Figure S1). On the other hand, employing ERA amplification significantly improved the sensitivity of EBV DNA detection using the CRISPR‒Cas12a system (Figure S1).

Secondly, the reaction conditions of ERA (such as primer, volume) were optimized to improve the system of ERA–Cas12a sensitivity and specificity.

Primer design is crucial for ERA. LMP2A transcripts are relatively stable and can be detected persistently in NPC and other EBV‐related malignant tumours. In total, we designed and tested 18 ERA primer pairs targeting the LMP‐2A gene of EBV. Of them, 12 primer pairs were tested for LMP1 fragments, with the most efficient amplification achieved using LMP1‐F2+R3 and LMP1‐F3+R3 (Figure S2A). Moreover, six primer pairs were tested for LMP2 fragments, with the most efficient amplification achieved using LMP2‐F3+R1 and LMP2‐F3+R2 (Figure S2B). The real‐time fluorescence curve demonstrated that LMP1‐F3+R3 and LMP2‐F3+R1 reached a plateau phase rapidly. Consequently, the primer pairs LMP1‐F3+R3 and LMP2‐F3+R1 were identified as the optimal choices for LMP1 and LMP2, respectively (Figure 1A,B).

**FIGURE 1:**
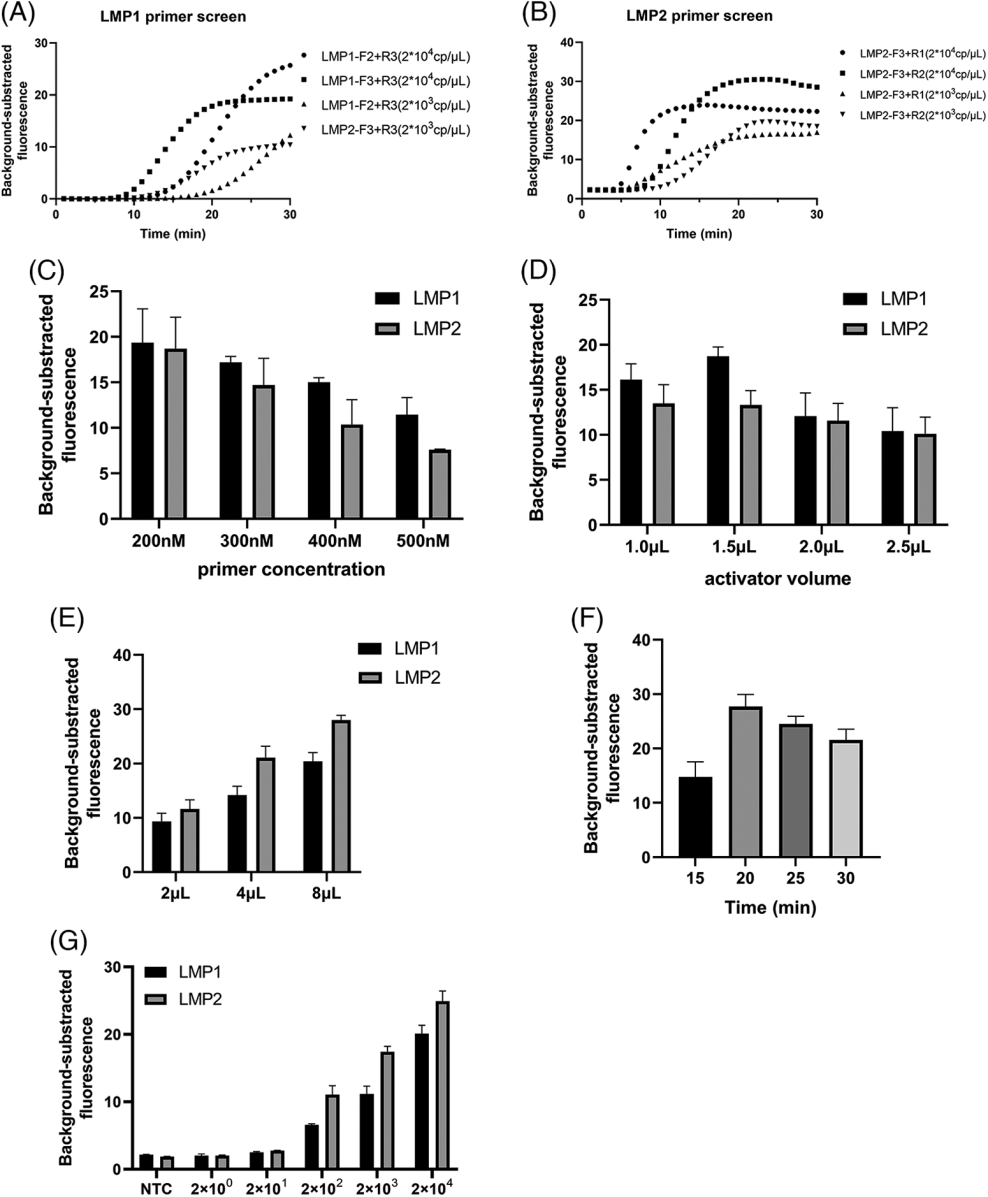
Optimization of ERA method. (A, B) Optimization of ERA primer, template concentration: 2 × 104 and 2 × 103 copies/µL. LMP1‐F3+R3 and LMP2‐F3+R1 were identified as the optimal primer pairs for LMP1 and LMP2 to proceed with further ERA optimization steps, respectively. (C) Comparison of different primer concentrations (200, 300, 400 and 500 nM) with template concentration = 2 × 103 copies/µL. (D) Comparison of different activator volumes (1, 1.5, 2.0 and 2.5 µL) with template concentration = 2 × 103 copies/µL. (E) Comparison of different template volumes (2, 4 and 8 µL) with template concentration = 2 × 103 copies/µL. (F) Measurement of background‐subtracted fluorescence at different ERA amplification times (15, 20, 25 and 30 min). (G) LOD of the ERA assay using 10‐fold diluted template concentrations (from 2 × 104 to 2 × 100 copies/µL).

Increasing primer pair concentrations resulted in a slower amplification curve with a decrease in fluorescence value. The fluorescence value was the highest with a primer concentration of 200 nM (Figure 1C), an activator volume of 1.5 µL (Figure 1D) and a template volume of 8 µL (Figure 1E). The optimal ERA time was 20 min (Figure 1F). These quantities were used for the subsequent experiments. These findings indicated that the limit of detection (LOD) of the fluorescence‐based ERA assay was 2 × 10^2^ copies/µL (Figure 1G). However, ERA alone could not detect four clinical EBV nucleic acid samples (Figure S3). EBV is a virus with double‐stranded DNA. Cas12a has the ability to identify target DNA in the presence of crRNA. While it mediates specific cleavage of target‐site sequences, Cas12a also exhibits non‐specific single‐stranded DNA (ssDNA) digestive activity once forming the Cas12a/crRNA/target DNA polymer, trigger the cleavage of nearby ssDNA fluorescent or other signal probes (called collateral cleavage characteristics). This characteristic has been increasingly developed and used for identifying various pathogens.

Next, the CRISPR–Cas12a system (concentration, buffer and probe) was optimized.

Screening out crRNAs with high specificity and efficiency was crucial for further testing. Among these crRNA candidates, we selected LMP1 crRNA3 and LMP2 crRNA1, with the strongest fluorescence signal, for their highest cleavage efficiency in CRISPR‒Cas12a/crRNA reaction (Figures 2A and S4A,B). To build the optimal reaction conditions, we adjusted various factors, such as Cas12a concentration, buffer type, buffer concentration, and F‐Q and F‐B probe concentrations. Template volume (6 µL) (Figure 2B), Cas12a concentration (50 nM) (Figure 2C) and crRNA concentration (180 nM) (Figure 2D), reaction buffer system NEBuffer 2.1 (Figure 2E) was selected. Three buffer concentrations (1×, 2× and 4×) were tested in this study. With NEBuffer 2.1, Cas12a activity peaked with the 1× buffer concentration (Figure S5).

**FIGURE 2:**
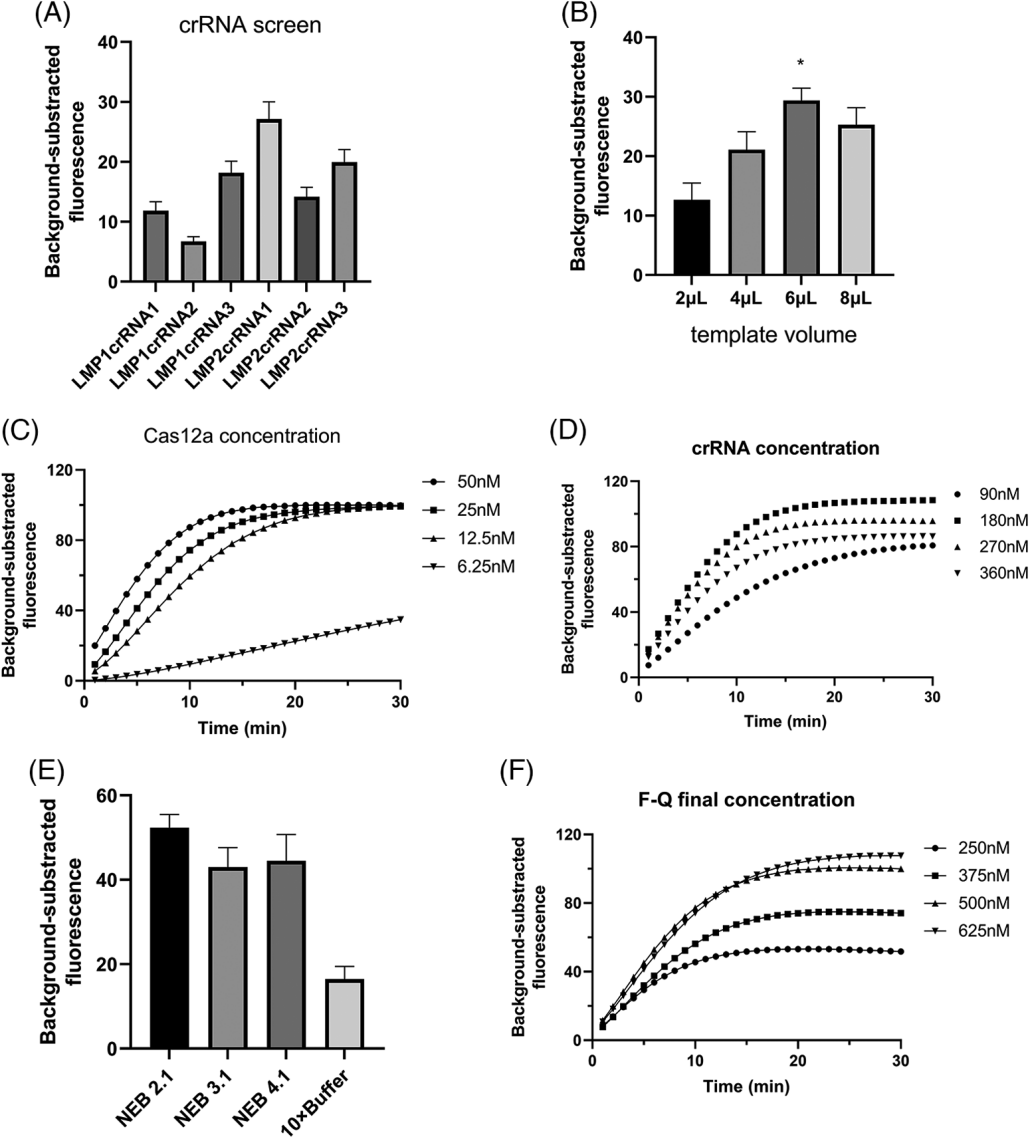
Optimization of CRISPR–Cas12a assay for ERA–Cas12a fluorescence system. (A–D) crRNA screening. Evaluation of background‐subtracted fluorescence using different crRNAs (A) and various template volumes (2, 4, 6 and 8 µL; B) at 30 min. (C, D) Real‐time fluorescence readouts at different Cas12a concentrations (6.25, 12.5, 25 and 50 nM; C) and different crRNA concentrations (90, 180, 270 and 360 nM; D). (E) Buffer selection. NEB 2.1, NEBuffer 2.1; NEB3.1, NEBuffer 3.1; NEB 4, NEBuffer 4. (F) Real‐time fluorescence readout at varying F‐Q reporter concentrations (250, 375, 500 and 625 nM). NTC, no target control.

The two ssDNA oligonucleotide types (TTATT and TTATTATT) and three ConRs extended to different lengths were used as reporters to optimize the F‐Q reporter (Table S3 and Figure S6A,B). In our study, the largest background‐subtracted fluorescence value was observed at F‐Q reporter concentration (500 nM) (Figure 2F).

The F‐B concentration (2.5 µM) (Figure 3B) and incubation time (30 min) (Figure 3C) were optimized for the ERA‐Cas12a lateral flow test. Following the optimization of CRISPR–Cas12a fluorescence system and ERA‐Cas12a lateral flow test, an integrated one‐tube ERA‐Cas12a reaction for detecting EB DNA was established. Figure 3A displays our ERA–Cas12a system workflow: EBV DNA is first isothermally amplified by the viral gene fragment EBV‐LMP‐2A. The ERA product is then recognized by the Cas12a‐crRNA complex, which triggers collateral cleavage activity, leading to ssDNA reporter cleavage. This may be followed by qualitative fluorescence or chromatographic detection of the cleavage. The optimized ERA‒Cas12a system could detect EBV as low as 20 copies/µL (Figures 3D and 4E), with a specificity of almost 100% but without cross‐reaction with other pathogens (Figure 4A,B).

**FIGURE 3:**
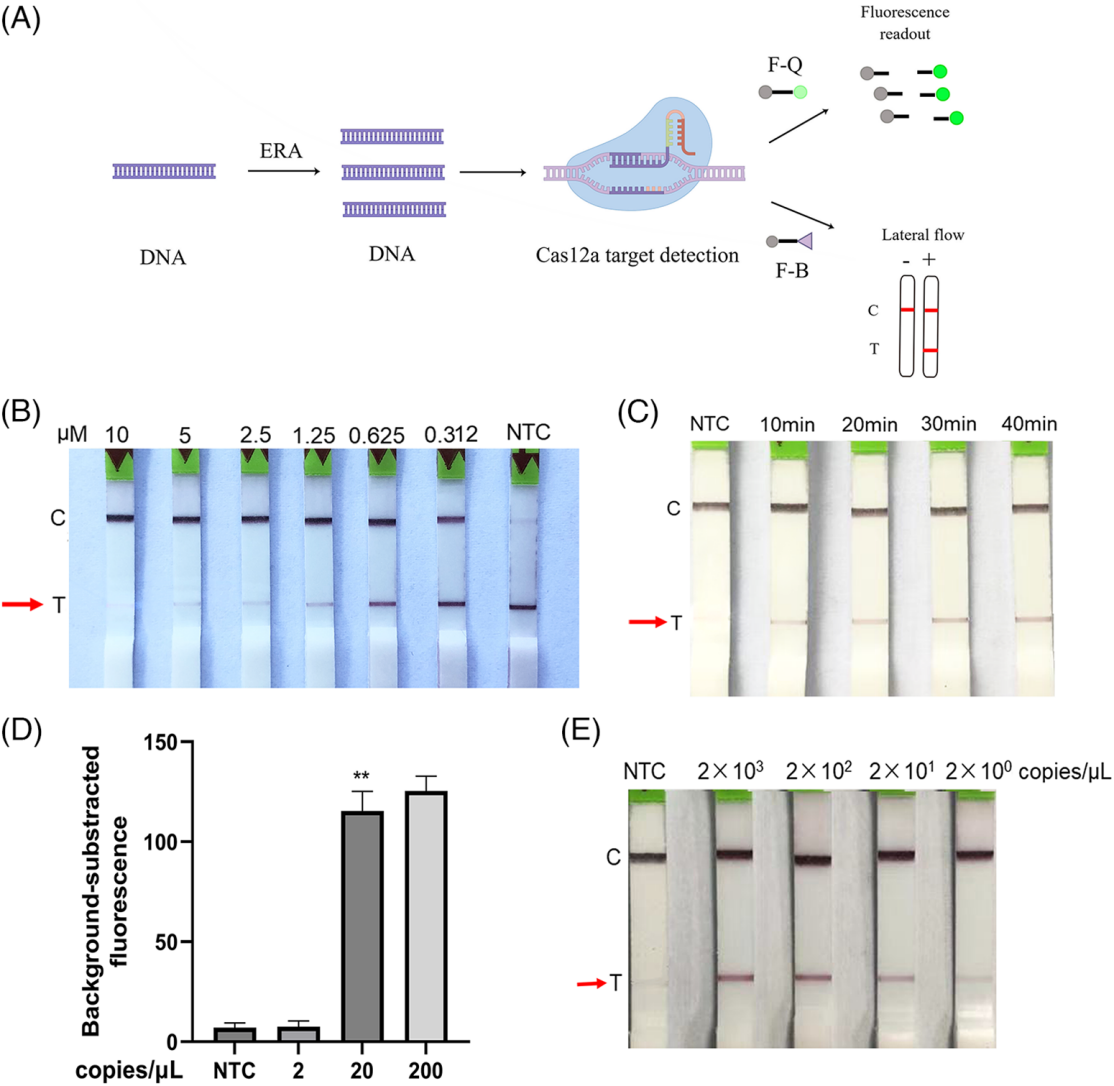
(A) Workflow of our ERA–Cas12a system. The workflow begins with the amplification of clinical EBV nucleic acid samples using the ERA technique, completed within 20 min. The amplified product is then targeted by the Cas12a‐crRNA complex, which activates collateral cleavage activity, resulting in the cleavage of an ssDNA reporter. Detection is achieved through qualitative methods by either observing the fluorescence signal or employing a chromatographic dipstick (T, test line; C, control line). This simple, efficient method affords a practical solution for the detection of EBV infections. (The workflow was drawn using Figdraw.) (B) Optimization of F‐B reporter concentration. Dipsticks were immersed in solutions with serial two‐fold dilutions of F‐B reporter (10, 5, 2.5, 1.25, .625 and .3125 µM). The optimal concentration was determined by identifying the lowest concentration that rendered the test line invisible. (C) Optimization of the incubation time of the CRISPR–Cas12a lateral‐flow system. The reaction tubes were incubated at 37°C for various durations (10, 20, 30 or 40 min). C, control band; NTC, no target control; T, test band. (D, E) The sensitivity of the ERA‒Cas12a assay for detecting EBV was evaluated using 10‐fold serial dilutions of the LMP2 template from 2 × 103 to 2 × 100 copies/µL. The detection was measured by background‐subtracted fluorescence readout (D) or lateral‐flow readout (E) at 30 min.

**FIGURE 4:**
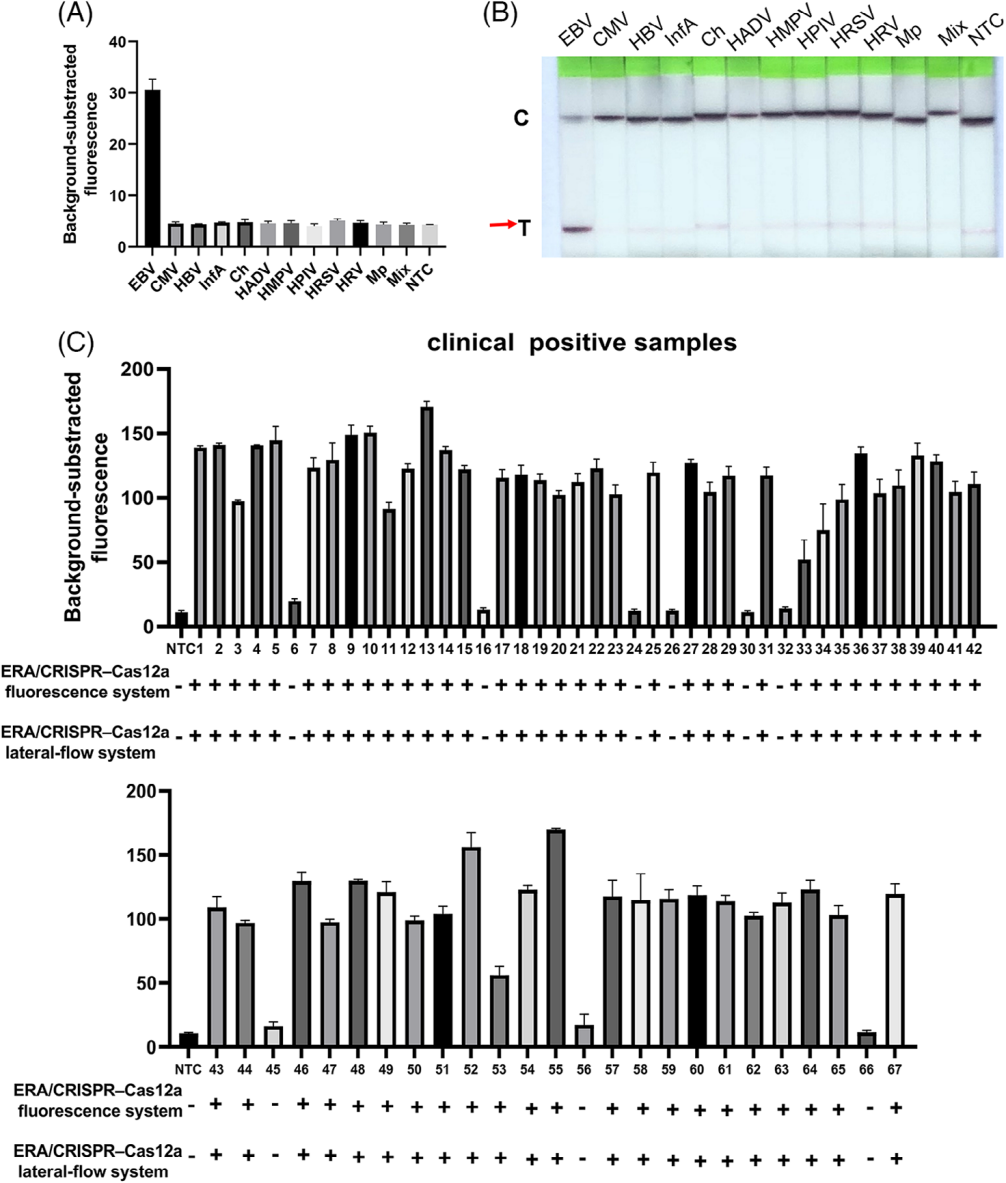
Verification of the specificity of ERA–Cas12a system. (A, B) Evaluation of the specificity of ERA–Cas12a assay for EBV detection. The assays to the nucleic acid samples extracted from various pathogens by the methods either fluorescence measurement with background subtraction (A) or a lateral‐flow readout (B). Boca, human bocavirus; Ch, Chlamydophila pneumoniae; CMV, cytomegalovirus; EBV, Epstein–Barr virus; HADV, human adenovirus; HBV, hepatitis B virus; HCOV, human coronaviruses; HMPV, human metapneumovirus, HPIV, human parainfluenza virus; HRSV, human respiratory syncytial virus; HRV, human rhinovirus; InfA, influenza A virus; InfB influenza B virus; Mp, Mycoplasma pneumoniae; Mix, mixed nucleic acid samples from the 13 Respiratory Pathogen Multiplex Detection Kit (Health Gene Tech. company) (respiratory pathogens including Boca, Ch, HADV, HCOV, HMPV, HPIV, HRV, InfA, InfB, 09H1N1, H3N2). (C) Confirmation of the accuracy of ERA–Cas12a system for EBV detection using clinical positive nucleic acid samples. The results were displayed as either the final fluorescence reading after 30 min (background‐subtracted fluorescence), or a lateral‐flow detection within 30 min. The fluorescence data obtained from ERA–Cas12a system were indicated in the upper section, while the results of the dual system assay were shown in the lower section. NTC, no target control; +, positive result; −, negative result.

Finally, subsequent validation of this one‐tube ERA‒Cas12a system with clinical EBV nucleic acid samples confirmed its sensitivity and specificity. Among 97 clinical samples evaluated, 58 out of 67 EBV‐positive samples returned positive results, while nine tested negative. Importantly, no false positives were observed in the 30 EBV‐negative samples (Figure 4C and Table S4). The combined ERA‐Cas12a fluorescence or lateral‐flow systems exhibited a positive predictive agreement of 86.6% and a negative predictive agreement of 100% when compared with qPCR detection methods (Table S5). Contrast to qPCR, ERA–Cas12a is a rapid, portable method for EBV detection and is more suitable for field testing.

In summary, our results offer an enhanced understanding of the factors affecting the sensitivity and efficiency of the ERA‒Cas12a system, which may facilitate its broader applications in nucleic acid detection. This system affords a rapid, convenient, inexpensive detection method for EBV nucleic acid detection, which may have clinical applicability for the screening and diagnosis of NPC and other EBV infection‐related diseases.

## AUTHOR CONTRIBUTIONS

ZH conceived the study and designed the experiments. JL, HC, XW, NC, LC, LD and FT conducted the experiments. JL, HC, XW, KL, DL and ZH analysed the data. JL, KL, DL and ZH wrote the paper. All authors contributed to drafting or revising the article, gave final approval of the version to be published and agree to be accountable for all aspects of the work.

## CONFLICT OF INTEREST STATEMENT

The authors have no conflict of interest to declare.

## ETHICS APPROVAL AND CONSENT TO PARTICIPATE

This investigation has been conducted in accordance with the ethical standards and according to the Declaration of Helsinki and according to national and international guidelines and has been approved by the institutional review board of the First People's Hospital of Chenzhou, Hunan, P.R. China.

## Supporting information



Supporting Information

## Data Availability

Data will be made available upon request.
