# Supplementary material for: Rapid, portable Epstein‒Barr virus DNA detection using enzymatic recombinase amplification combined with the CRISPR–Cas12a system
Source: Clin Transl Med. 2024 Sep 23;14(9):e70028. doi: 10.1002/ctm2.70028 (PMC11420463; doi:10.1002/ctm2.70028)
Supplement: Supplementary file 1 — Supporting Information [file CTM2-14-e70028-s001.docx]

**Supplementary materials**

1. **Materials and methods**
   1. **Target sequences, materials, and reagents**

Epstein-Barr virus (EBV) is a human herpesvirus that commonly infects most people. While most EBV infections are asymptomatic, the virus itself is associated with epithelioid and lymphoid malignancies. EBV LMP-2A (EBV latent membrane protein 2A), is a 497-amino acid EBV protein that is present on the surface of EBV-infected cells and is expressed in most EBV-related malignancies(36). The EBV-LMP-2A gene sequence was queried via NCBI. Two DNA sequences EBV-LMP-2A-1 (LMP1) and EBV-LMP-2A-2 (LMP2), which could be used for designing ERA primers and contained Cas12a PAM sites, were identified. These sequences were cloned and inserted into the plasmid vector pUC57 from GenScript Biotech Corporation (Nanjing, China); the sequences were used as templates for subsequent experiments (Table S1). The NEB Buffer, HiScribe T7 High Yield RNA Synthesis Kit (E2040S), and LbCas12a were purchased from NEB (New England Biolabs, MA, USA), and the Enzymatic Recombinase Amplification Kit (KS101/KS103) was procured from GenDx Biotech Corporation (Suzhou, China).

- 1. **ERA primer selection and CRISPR RNA transcription**

According to the principles of primer design, the primers for ERA were selected using the Primer-BLAST online tool (<https://www.ncbi.nlm.nih.gov/tools/primer-blast/>). BLAST was used to exclude cross reactivity of the primers with nucleic acid sequences from other pathogens and sources. Four forward primers, three reverse primers, and one PB1 probe for LMP1, as well as three forward primers, two reverse primers, and a PB2 probe for LMP2, were selected (Table S2). In total, 18 primer pairs were tested, and the most efficient combinations were selected through fluorescence-based ERA.

Three CRISPR RNAs (crRNAs) were designed for each target sequence using CRISPR-DT. The templates used for each crRNA transcription were annealed by two overlapping oligonucleotide sequences. One oligonucleotide sequence contained the T7 promoter sequence, and the other contained a spacer sequence (37). The annealed product was incubated with T7 RNA polymerase overnight at 37°C to transcribe into crRNA; next, crRNA was purified using an miRNeasy Micro Kit (cat 217084; Qiagen, Hilden, Germany). Table S3 presents the template sequences used for crRNA transcription.

- 1. **ERA assay and optimization**

The reaction system for this method comprised ERA Basic (20 μL), ddH_2_O (21 μL), 10 μM forward primer (2 μL), 10 μM reverse primer (2 μL), 5 μM fluorescence probe (1 μL), and template (2 μL). The mixture was vortexed, briefly centrifuged, and then added to a reaction tube. An activator (2 μL) was added to the tube cap, which was then used to close the tube; this was followed by centrifugation for a few seconds. The tubes were incubated in a LightCycler 480 II Quantitative PCR (Hoffmann-La Roche Ltd, Bermuda) at 37°C for 30 min, and the fluorescence values were acquired every 1 min.

To ensure the accuracy of the results, the primer pairs with poor amplification efficiency were first removed using a solution with 2 × 10^4^ copies/μL plasmid as the template. Subsequently, lower plasmids concentrations (2 × 10^3^ copies/μL) were used to screen for primer pairs with good amplification efficiency (i.e., fast peak onset and high fluorescence values).

The primer concentration (200, 300, 400, and 500 nM), activator volume (1, 1.5, 2, and 2.5 μL), template volume (2, 4, and 8 μL), and reaction times (15, 20, 25, and 30 min) were optimized for the ERA system.

The plasmid DNA copy number was calculated according to the following formula: DNA copy number = DNA mass concentration/ (Number of bp × Relative molecular mass of one bp) × 6.02 × 10^23^.The solution of the calculated DNA copy number was subjected to 10-fold serial to the concentrations from 2 × 10^0^ to 2 × 10^4^ copies/μL. Subsequently, the prepared standards were stored at -20 ℃ until use. The limit of detection (LOD) was tested with tenfold serially diluted template and is the lowest analyte concentration at which detection is feasible. The LOD of the assay was validated with three replicates.

- 1. **CRISPR–Cas12a-mediated fluorescence cleavage assay**

In the CRISPR‒Cas12a cleavage assay, an optimal plasmid template concentration of 200 copies/μL was used, with the ERA product as a template. The reaction system components were as follows: 2 μL of buffer, 13 μL of DEPC H_2_O, 1 μL of Cas12a (1 μM), 1 μL of crRNA (5 μM), 1 μL of ssDNA F-Q reporter (5 μM), and 2 μL of template DNA. The Cas12a system was mixed thoroughly with the ERA product and incubated at 37°C for 30 min. The fluorescence values were collected every minute, and the transcleavage activity was monitored in real-time on the LightCycler 480 II Quantitative PCR (Hoffmann-La Roche Ltd, Bermuda).

CrRNA with good cleavage efficiency was then screened for; moreover, the Cas12a concentration (6.25, 12.5, 25, and 50 nM), crRNA concentration (90, 180, 270, and 360 nM), buffer type (NEBuffer 2.1, NEBuffer 3.1, NEBuffer 4, and 10× buffer), template dosage (2, 4, 6, and 8 μL), and F-Q reporter concentration (250, 375, 500, and 625 nM) involved in the Cas12a reaction were optimized. By using this optimized Cas12a-based assay, we could detect the presence of ERA products more effectively and efficiently.

- 1. **CRISPR–Cas12a-mediated** **lateral-flow cleavage assay**

Similar to the CRISPR–Cas12a-mediated fluorescence cleavage assay (which requires an F-Q reporter for reaction monitoring), the CRISPR‒Cas12a lateral-flow system depends on an F-B reporter to indicate the results. After the reaction, 80 μL of HybriDetect assay buffer was added to the tube, followed by thorough mixing. Next, HybriDetect strips (Milenia Biotech, Gießen, Germany) were inserted, and the results were observed after 5 min.

To prevent the hook effect due to inappropriate concentrations, the F-B reporter concentration required optimization. In contrast to the real-time transcleavage monitoring used for the fluorescence system, the optimal reaction time of the CRISPR–Cas12a lateral-flow assay was determined. This was followed by formal analysis such that it did not affect detection accuracy.

- 1. **Summary of the one-tube detection method**

The proposed method integrated ERA and Cas12a cleavage in a one-tube reaction system. First, 24µL of the ERA system containing template was added into the tube, and activator (1µL) was added to the tube cap. Second, centrifuge the tube for a few seconds and replace it with a new tube cap. Third, 35µL of Cas12a cleavage system was added to the tube cap, and the whole tube was placed into water bath for 20 min to complete ERA. Finally, mixing of CRISPR reagent and amplicons was completed by centrifuging the tube, and the whole tube was incubated in a constant temperature environment (LightCycler 480 II Quantitative PCR for fluorescence assay and water bath for lateral-flow assay) for 30 min to perform the CRISPR reaction.

- 1. **Validation with clinical nucleic acid samples**

The collection and use of clinical nucleic acid samples was approved by The Ethics Committee of the First People's Hospital of Chenzhou (approval number:2019015), and all patients provided informed consent. Blood samples from patients with suspected EBV infection were processed at the Department of Clinical Laboratory, the First People's Hospital of Chenzhou. DNA was extracted from blood samples using a nucleic acid extraction kit (Tianlong, Xian, China) according to the manufacturer’s instructions. DNA extracts were initially used for molecular diagnosis of EBV by qPCR using Epstein-Barr virus nucleic acid detection kit (Sansure Biotech Inc, Hunan, China). Excess DNA extracts from these samples were then used and stored at −20℃, without any personally identifiable information being collected, for validation of ERA-CRISPR/Cas12a diagnostics detection of EBV.

To assess the specificity combined using ERA with CRISPR‒Cas12a for EBV detection, the clinical nucleic acid samples of EBV, Cytomegalovirus (CMV), hepatitis B virus (HBV), influenza A virus (InfA), *Chlamydophila pneumoniae* (Ch), human adenovirus (HADV), human metapneumovirus (HMPV), human parainfluenza virus (HPIV), human respiratory syncytial virus (HRSV), human rhinovirus (HRV), human bocavirus (Boca), and *Mycoplasma pneumoniae* (Mp) that identified in Department of Clinical Laboratory, the First People's Hospital of Chenzhou by Commercialized reagent kits, were collected and used to detect by this method.

To assess the sensitivity of this method, in total, 30 EBV DNA-negative and 67 EBV DNA-positive nucleic acid samples determined through clinical real-time qPCR kit (Sansure Biotech Inc, Hunan, China) were reanalyzed using the one-tube ERA/CRISPR‒Cas12a system.

- 1. **Statistical analysis**

Data are expressed as means ± standard deviations (SDs). Fluorescence readouts were reported as background-subtracted values, whereby the values measured before reaction progression were subtracted from the observed fluorescence values. Data panels were generated using GraphPad Prism (version 9).

Fig. S1

**
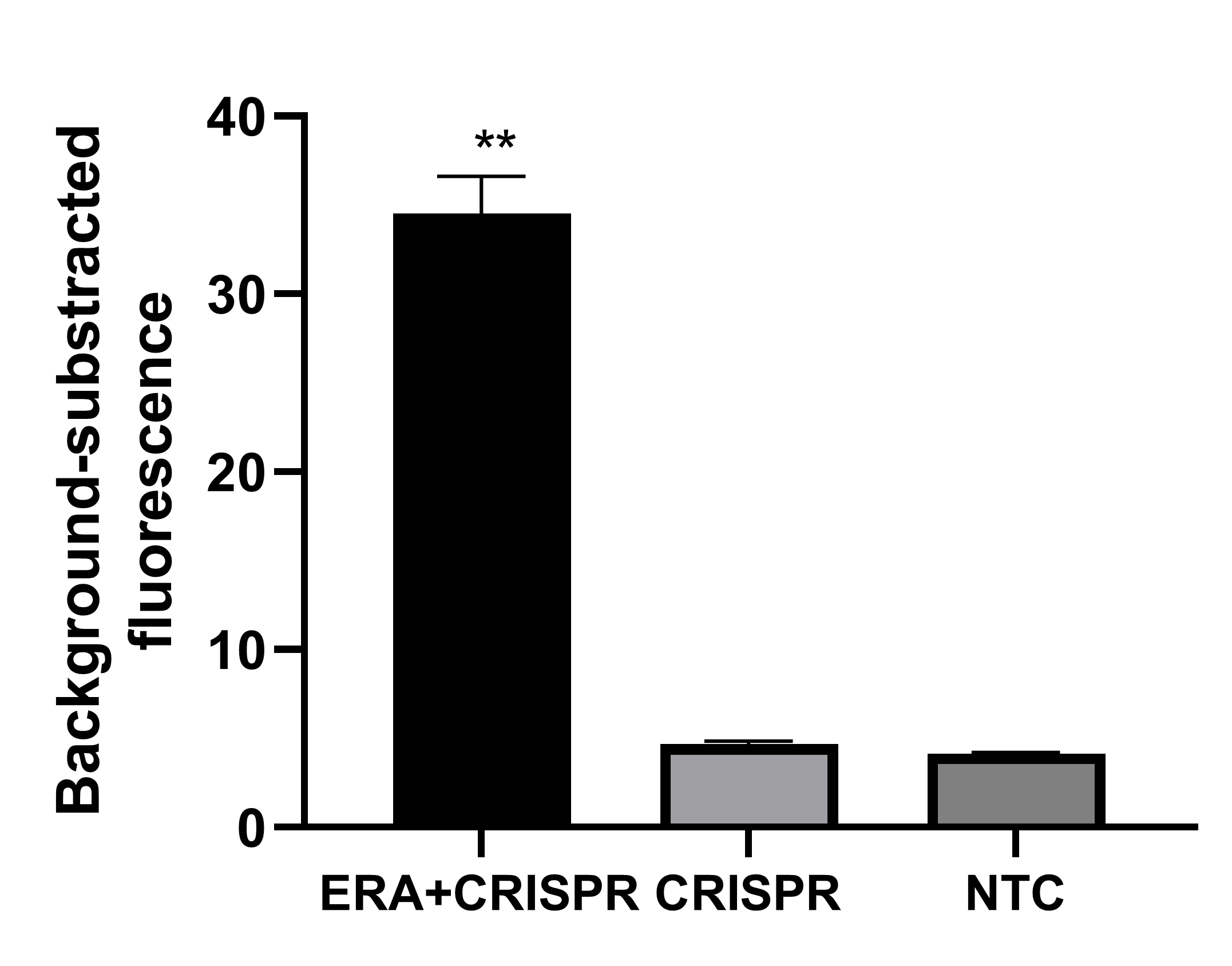
**

**Supplementary Figure 1. The importance of ERA for ERA combined CRISPR/Cas12a system.**

The importance of ERA for ERA combined CRISPR/Cas12a system. ERA + CRISPR: samples were amplified by ERA and then tested using a CRISPR system; CRISPR: samples were tested directly using the CRISPR system without ERA amplification.

Fig. S2


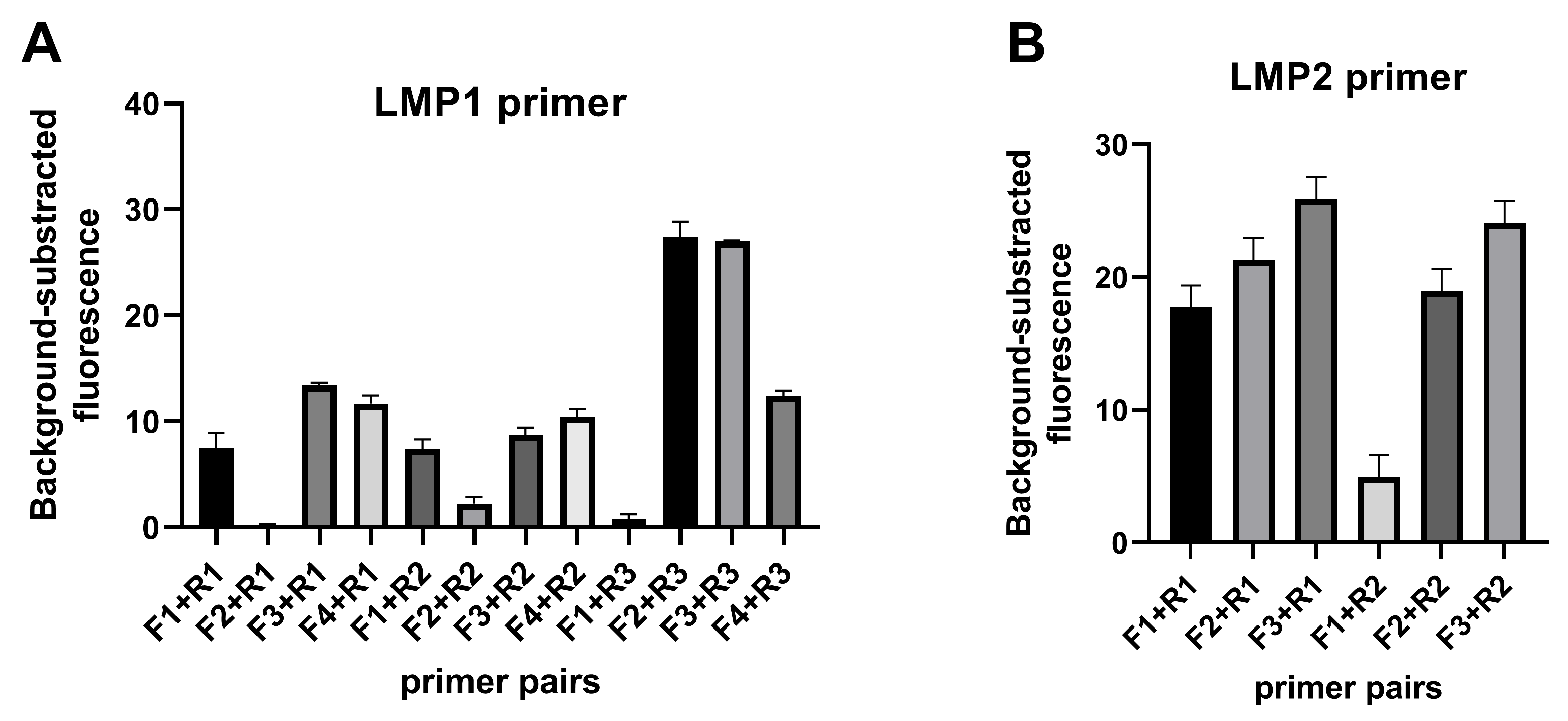


**Supplementary Figure 2. Optimization of ERA assay** (related to Fig.2).

Screening of ERA primer pairs for EBV, the template concentration for ERA was 2×10^4^copies/μL. A total of eighteen ERA-primer pairs targeting the LMP-2A genes (LMP1 and LMP2) of EBV were designed and tested. (A) Twelve primer pairs were tested for LMP1 fragments, with the most efficient amplification achieved using LMP1-F2+R3, and LMP1-F3+R3. (B) For LMP2 fragments, six primer pairs were tested, with the most efficient amplification achieved using LMP2-F3+R1 and LMP2-F3+R2.

Fig. S3


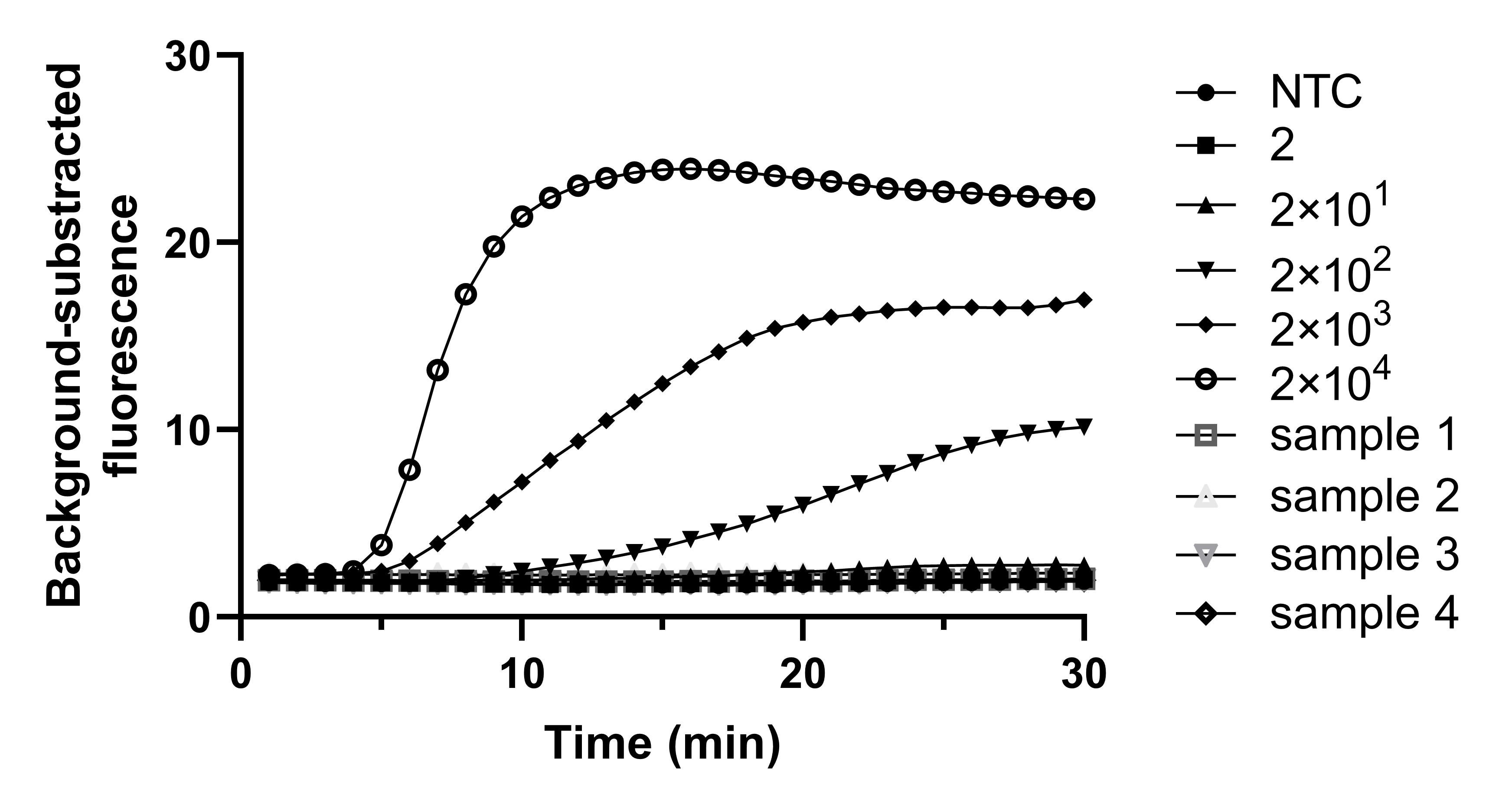
 **Supplementary Figure 3. Real-time fluorescence curves of LOD of ERA** (related to Fig.2).

Using the optimized reaction conditions of 200 nM LMP2-F3+R1 primer, 1.5μL activator volume, and 8 μL template volume, the minimum detection limit (LOD) of the ERA reaction was tested with ten-fold serial diluted template and four clinical samples. NTC, negative test control; 2, 2×10^1^,2×10^2^,2×10^3^,2×10^4^, indicate different concentration targeted templates; sample1,2,3, and 4, indicate four EBV nucleic acid templates.

Fig. S4


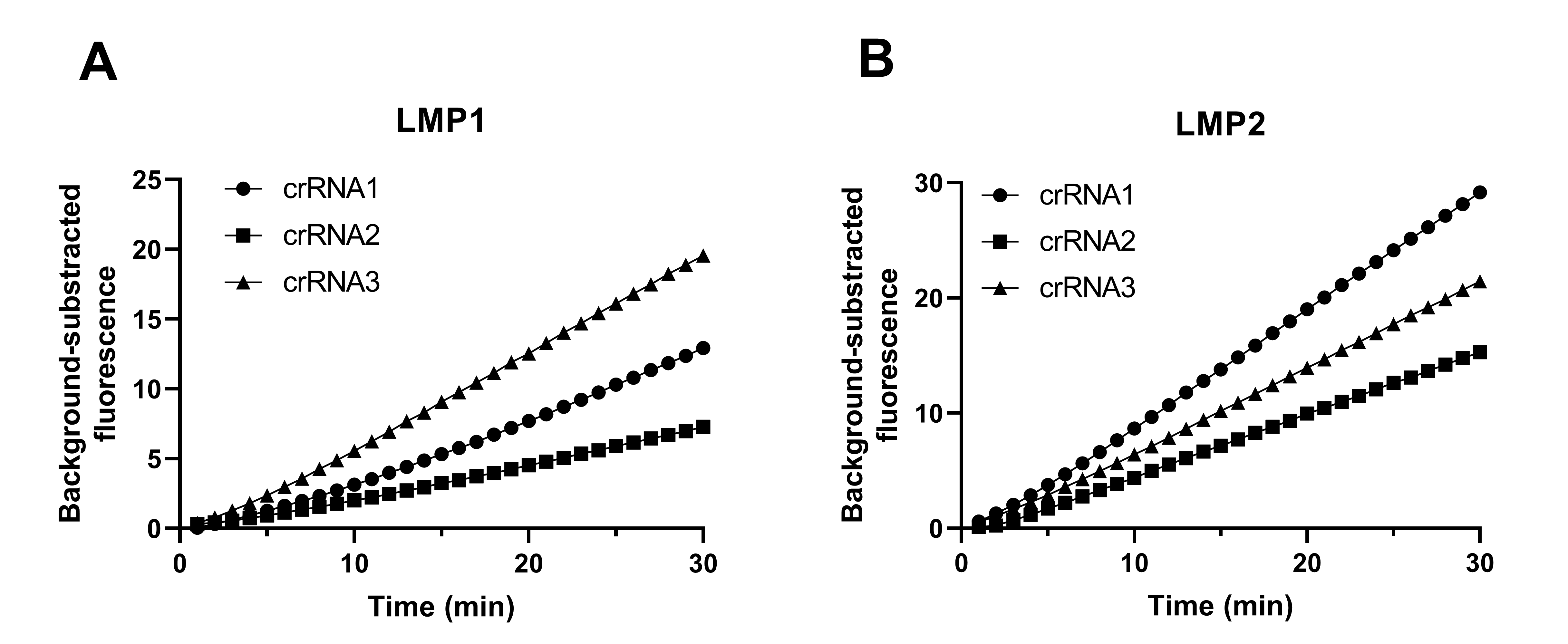


**Supplementary Figure 4. crRNAs screening for targeting EBV LMP1 and LMP2** (related to Fig.3) Real-time fluorescence curves of crRNAs to target LMP1 gene (A) and LMP2 gene (B).

Fig. S5


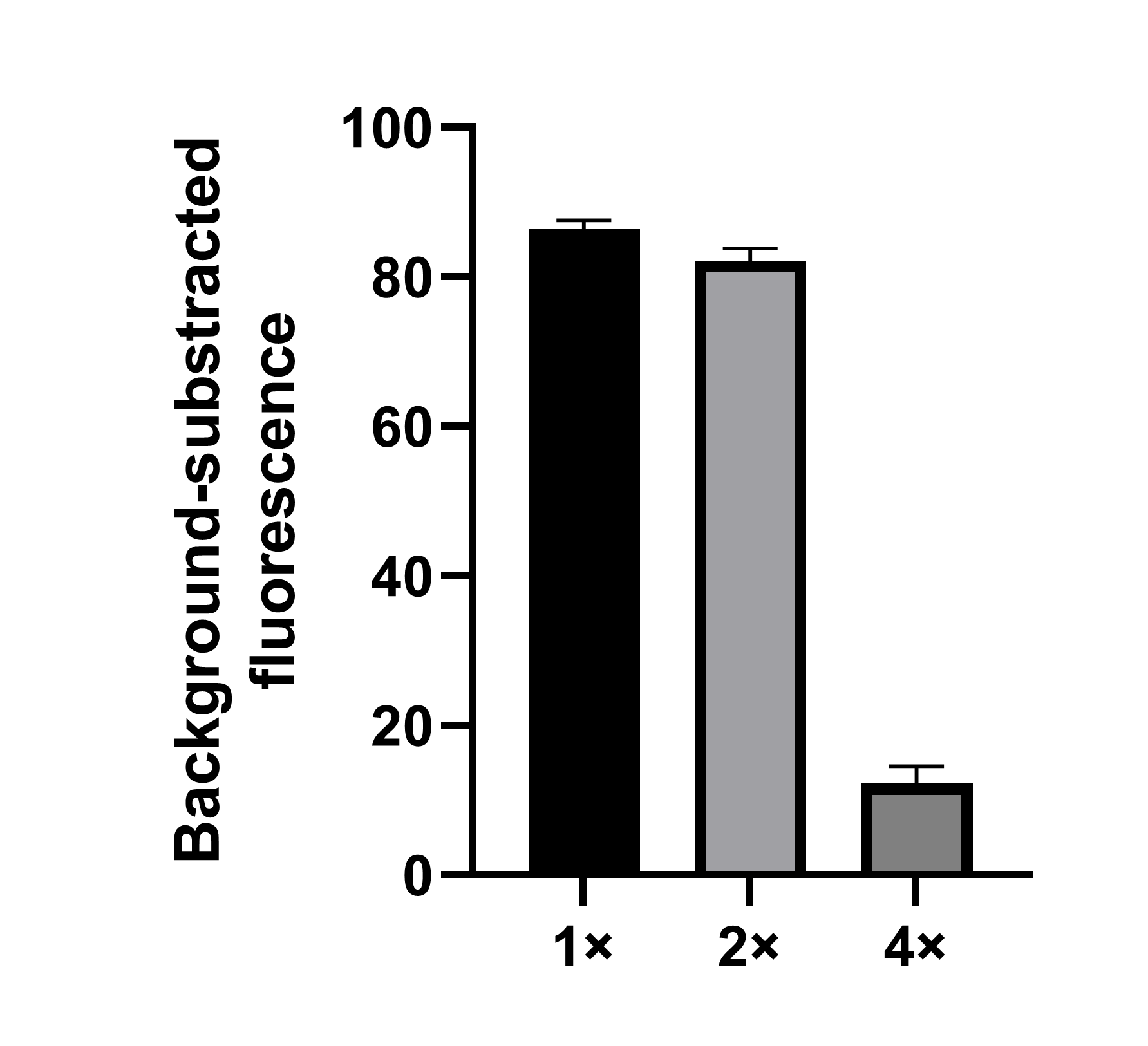


**Supplementary Figure 5. Effect of highly concentrated buffers on Cas12a trans-cleavage activity** (related to Fig.3)**.**

1×,2×,4×, three kinds of concentrated buffers (NEBbuffer 2.1).

Fig. S6


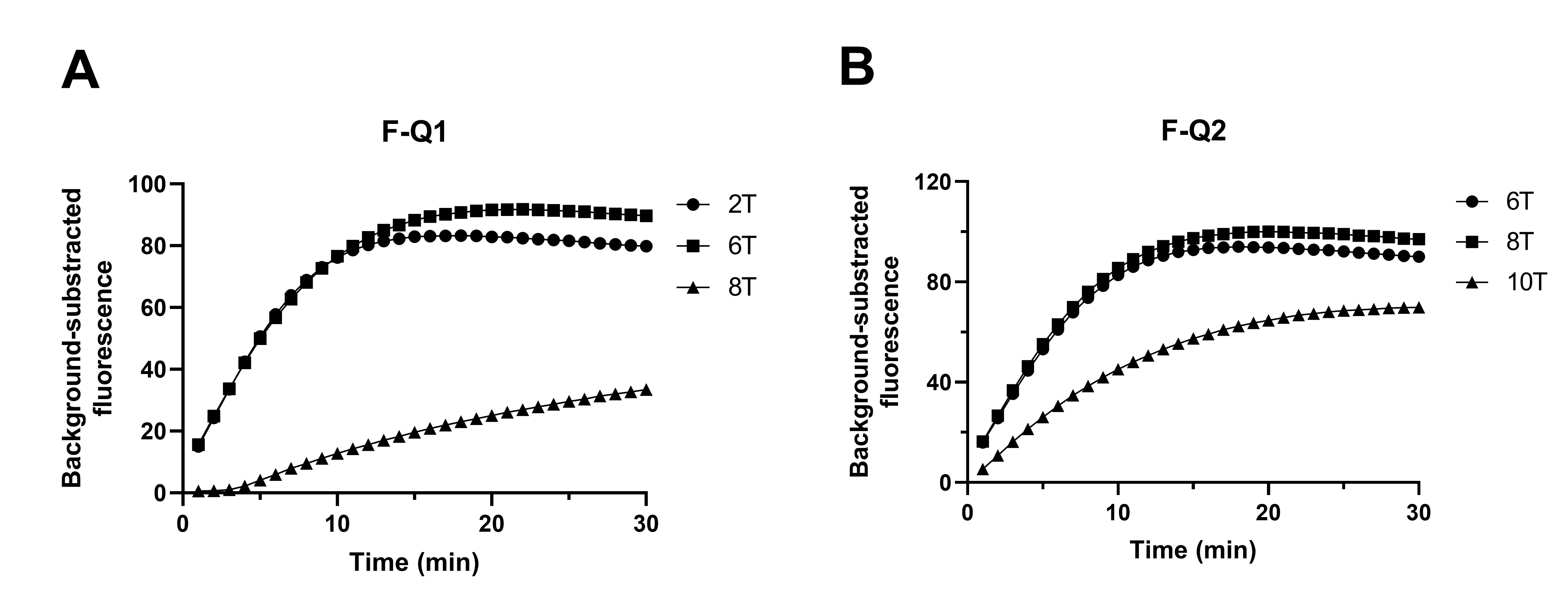


**Supplementary Figure 6. Real-time fluorescence curves of three different lengths of two types of ssDNA reporter** (related to Fig.3)**.**

A. The fluorescence of three different lengths of F-Q1 report. F-Q1, 5-TTATTATT-3’; 2T, F-Q1-2T;6T, F-Q1-6T; 8T, F-Q1-8T. B. The fluorescence of three different lengths of F-Q2 report. F-Q2, 5’-TTATT-3’;6T, F-Q2-6T;8T, F-Q2-8T;10T, F-Q2-10T.

**Table S1 Target sequences of EBV LMP1&LMP2**

| Name | Sequences(5’→3’) |
| --- | --- |
| LMP1 | caagctgtgggaatgcggtggccaagtgcaacaggaaatggaaaggcagtgcggcaatcagaagggggagtgcgtagtgttgtgggaagcggcagtgtaatctgcacaaagaggcgcggggcgcgcaacgttgggaggtcgttggcggcaggcgggaggccgtgctttaggggggttcaggtgaggcaaggctgtggggtaaccgtaggggaggcgggtgaggcggctaagagggctaagggtcggcgggtgacgaagcagcagacggcggatatgggaatttcagaatgaggtggcggattcaggcgaaaagggtgtgggctgtgcgagtgtcatgaggcaggcgcggaaagtcgctgcggcttgctggggcatggggggccgcgcattcctggaaaaagtggagggggcgtggcctt |
| LMP2 | ggcggagagtagcgtagaatccagccagtggtctacccggtcgcatggtggcttcttagatgaggagcaggcataaaagtccaaacaggacacagagtaccaccaggagtagtcttagtctgctgacgtctgggtcctcggggcaggggtggctaggcctggtctccgtagaagagccgggcaggccgcaggcagaggactgctgctctagcaaagcacgctccaggacgtgtaccatctcgagagtgaggcacagctgttttcgtggacttttatacagtaaggacaaggaaagaaggccagaggaatgtggaaagatgagcgaggacaggtgtggaggttttgggctagctcttagtttctgggtgtgagagagggattaaagtgcttatgcgcaaagaatgtgtcaacaacaggtgttcctgcctctgctggcatgagttaggtgtggcttgggctgaatccaaatgtgtattggcacaagatggaaagcaaagttgctggagttact |

**Table S2 Sequences of primers and probes used in this study**

| Name | Sequences(5’→3’) |
| --- | --- |
| LMP1-F1 | AAATGGAAAGGCAGTGCGGCAATCAGAAG |
| LMP1-F2 | AATCAGAAGGGGGAGTGCGTAGTGTTGTG |
| LMP1-F3 | CGTAGTGTTGTGGGAAGCGGCAGTGTAAT |
| LMP1-F4 | GGTGAGGCAAGGCTGTGGGGTAACCGTAG |
| LMP1-R1 | ACAGCCCACACCCTTTTCGCCTGAATCCG |
| LMP1-R2 | ATGACACTCGCACAGCCCACACCCTTTTC |
| LMP1-R3 | ATGCCCCAGCAAGCCGCAGCGACTTTCCG |
| LMP2-F1 | GATGAGGAGCAGGCATAAAAGTCCAAACAG |
| LMP2-F2 | CCAAACAGGACACAGAGTACCACCAGGAGT |
| LMP2-F3 | ACACAGAGTACCACCAGGAGTAGTCTTAGT |
| LMP2-R1 | TAAGCACTTTAATCCCTCTCTCACACCCAG |
| LMP2-R2 | TGGATTCAGCCCAAGCCACACCTAACTCAT |
| PB1 | GTGACGAAGCAGCAGACGGCGGATATGGGAA(FAM-dT) (THF) (BHQ1-dT) CAGAATGAGGTGG(C3-SPACER) |
| PB2 | CAGTAAGGACAAGGAAAGAAGGCCAGAGGAA(FAM-dT) (THF) (BHQ1-dT) GGAAAGATGAGCG(C3-SPACER) |

**Table S3 Sequences of crRNA and ssDNA F-Q reporter**

| Name | Sequences(5’→3’) |
| --- | --- |
| LMP1-crRNA 1 | UAAUUUCUACUAAGUGUAGAUAGAAUGAGGUGGCGGAUUCAGGC |
| LMP1-crRNA 2 | UAAUUUCUACUAAGUGUAGAUGCCUGAAUCCGCCACCUCAUUCU |
| LMP1-crRNA 3 | UAAUUUCUACUAAGUGUAGAUCGCGCCUGCCUCAUGACACUCGC |
| LMP2-crRNA 1 | UAAUUUCUACUAAGUGUAGAUCUAGAGCAGCAGUCCUCUGCCUG |
| LMP2-crRNA 2 | UAAUUUCUACUAAGUGUAGAUGGCUAGCUCUUAGUUUCUGGGUG |
| LMP2-crRNA 3 | UAAUUUCUACUAAGUGUAGAUAUCCCUCUCUCACACCCAGAAAC |
| F-Q1-2T | TTATTATT |
| F-Q1-6T | TTTTTTATTATT |
| F-Q1-8T | TTTTTTTTATTATT |
| F-Q1-10T | TTTTTTTTTTATTATT |
| F-Q2-6T | TTTTTTATT |
| F-Q2-8T | TTTTTTTTATT |
| F-Q2-10T | TTTTTTTTTTATT |

**Table S4 The detected results of nucleic acid samples**

| No. | Results of qPCR Diagnostics | copy number in qPCR testing(copies/mL) | EBV test by ERA/CRISPR-Cas12a based fluorescence | EBV test by ERA/CRISPR-Cas12a based lateral-flow | Notes |
| --- | --- | --- | --- | --- | --- |
| 1 | EBV + | 3.3*10^2^ | + | + |  |
| 2 | EBV + | 1.8*10^4^ | + | + |  |
| 3 | EBV + | 6.7*10^2^ | + | + |  |
| 4 | EBV + | 3.2*10^2^ | + | + |  |
| 5 | EBV + | 6.7*10^2^ | + | + |  |
| 6 | EBV + | 1.8*10^2^ | - | - |  |
| 7 | EBV + | 5.7*10^2^ | + | + |  |
| 8 | EBV + | 1.3*10^4^ | + | + |  |
| 9 | EBV + | 2.7*10^2^ | + | + |  |
| 10 | EBV + | 8.8*10^3^ | + | + |  |
| 11 | EBV + | 1.9*10^3^ | + | + |  |
| 12 | EBV + | 5.1*10^2^ | + | + |  |
| 13 | EBV + | 5.7*10^3^ | + | + |  |
| 14 | EBV + | 4.3*10^2^ | + | + |  |
| 15 | EBV + | 4.0*10^3^ | + | + |  |
| 16 | EBV + | 2.2*10^2^ | - | - |  |
| 17 | EBV + | 1.2*10^4^ | + | + |  |
| 18 | EBV + | 6.8*10^2^ | + | + |  |
| 19 | EBV + | 2.6*10^3^ | + | + |  |
| 20 | EBV + | 3.1*10^3^ | + | + |  |
| 21 | EBV + | 8.5*10^2^ | + | + |  |
| 22 | EBV + | 8.7*10^2^ | + | + |  |
| 23 | EBV + | 8.0*10^3^ | + | + |  |
| 24 | EBV + | 1.4*10^2^ | - | - |  |
| 25 | EBV + | 5.2*10^2^ | + | + |  |
| 26 | EBV + | 1.6*10^2^ | - | - |  |
| 27 | EBV + | 2.2*10^2^ | + | + |  |
| 28 | EBV + | 8.3*10^4^ | + | + |  |
| 29 | EBV + | 5.4*10^3^ | + | + |  |
| 30 | EBV + | 2.3*10^2^ | - | - |  |
| 31 | EBV + | 3.0*10^4^ | + | + |  |
| 32 | EBV + | 2.1*10^2^ | - | - |  |
| 33 | EBV + | 8.1*10^2^ | + | + |  |
| 34 | EBV + | 7.7*10^2^ | + | + |  |
| 35 | EBV + | 7.4*10^2^ | + | + |  |
| 36 | EBV + | 4.1*10^3^ | + | + |  |
| 37 | EBV + | 4.1*10^3^ | + | + |  |
| 38 | EBV + | 3.6*10^2^ | + | + |  |
| 39 | EBV + | 6.2*10^3^ | + | + |  |
| 40 | EBV + | 9.1*10^3^ | + | + |  |
| 41 | EBV + | 1.6*10^3^ | + | + |  |
| 42 | EBV + | 1.8*10^3^ | + | + |  |
| 43 | EBV + | 7.4*10^2^ | + | + |  |
| 44 | EBV + | 1.1*10^3^ | + | + |  |
| 45 | EBV + | 1.6*10^2^ | - | - |  |
| 46 | EBV + | 8.5*10^2^ | + | + |  |
| 47 | EBV + | 6.7*10^2^ | + | + |  |
| 48 | EBV + | 8.3*10^2^ | + | + |  |
| 49 | EBV + | 1.2*10^3^ | + | + |  |
| 50 | EBV + | 9.2*10^2^ | + | + |  |
| 51 | EBV + | 2.1*10^3^ | + | + |  |
| 52 | EBV + | 4.5*10^4^ | + | + |  |
| 53 | EBV + | 7.3*10^2^ | + | + |  |
| 54 | EBV + | 2.4*10^3^ | + | + |  |
| 55 | EBV + | 5.6*10^4^ | + | + |  |
| 56 | EBV + | 1.2*10^2^ | - | - |  |
| 57 | EBV + | 1.4*10^3^ | + | + |  |
| 58 | EBV + | 8.1*10^2^ | + | + |  |
| 59 | EBV + | 7.7*10^2^ | + | + |  |
| 60 | EBV + | 7.4*10^2^ | + | + |  |
| 61 | EBV + | 1.1*10^3^ | + | + |  |
| 62 | EBV + | 1.3*10^3^ | + | + |  |
| 63 | EBV + | 8.6*10^2^ | + | + |  |
| 64 | EBV + | 9.2*10^2^ | + | + |  |
| 65 | EBV + | 9.1*10^2^ | + | + |  |
| 66 | EBV + | 2.0*10^2^ | - | - |  |
| 67 | EBV + | 1.4*10^3^ | + | + |  |
| 68 | EBV - | <100 | - | - |  |
| 69 | EBV - | <100 | - | - |  |
| 70 | EBV - | <100 | - | - |  |
| 71 | EBV - | <100 | - | - |  |
| 72 | EBV - | <100 | - | - |  |
| 73 | EBV - | <100 | - | - |  |
| 74 | EBV - | <100 | - | - |  |
| 75 | EBV - | <100 | - | - |  |
| 76 | EBV - | <100 | - | - |  |
| 77 | EBV - | <100 | - | - |  |
| 78 | EBV - | <100 | - | - |  |
| 79 | EBV - | <100 | - | - |  |
| 80 | EBV - | <100 | - | - |  |
| 81 | EBV - | <100 | - | - |  |
| 82 | EBV - | <100 | - | - |  |
| 83 | EBV - | <100 | - | - |  |
| 84 | EBV - | <100 | - | - |  |
| 85 | EBV - | <100 | - | - |  |
| 86 | EBV - | <100 | - | - |  |
| 87 | EBV - | <100 | - | - |  |
| 88 | EBV - | <100 | - | - |  |
| 89 | EBV - | <100 | - | - |  |
| 90 | EBV - | <100 | - | - |  |
| 91 | EBV - | <100 | - | - |  |
| 92 | EBV - | <100 | - | - |  |
| 93 | EBV - | <100 | - | - |  |
| 94 | EBV - | <100 | - | - |  |
| 95 | EBV - | <100 | - | - |  |
| 96 | EBV - | <100 | - | - |  |
| 97 | EBV - | <100 | - | - |  |
| 98 | CMV |  | - | - | ^*^ |
| 99 | HBV |  | - | - | ^*^ |
| 100 | InfA |  | - | - | ^*^ |
| 101 | Ch |  | - | - | ^*^ |
| 102 | HADV |  | - | - | ^*^ |
| 103 | HMPV |  | - | - | ^*^ |
| 104 | HPIV |  | - | - | ^*^ |
| 105 | HRSV |  | - | - | ^*^ |
| 106 | HRV |  | - | - | ^*^ |
| 107 | Mp |  | - | - | ^*^ |

^*^ CMV, Cytomegalovirus; HBV, hepatitis B virus; InfA, influenza A virus; Ch, Chlamydophila pneumoniae; HADV, human adenovirus; HMPV, human metapneumovirus, HPIV, human parainfluenza virus; HRSV, human respiratory virus; HRV, human rhinovirus; Mp, *Mycoplasma pneumoniae*. +, positive; -, negative.

**Table S5** Our ERA/CRISPR–Cas12a system versus a commercial qPCR kit

| EBV | ERA/CRISPR–Cas12 Fluorescence System | | ERA/CRISPR–Cas12 Dipstick System | | Total  (*N* = 97) |
| --- | --- | --- | --- | --- | --- |
|  | Positive | Negative | Positive | Negative |  |
| Positive | 58 | 9 | 58 | 9 | 67 |
| Negative | 0 | 30 | 0 | 30 | 30 |
| PPA/NPA | PPA:86.6% | NPA:100% | PPA: 86.6% | NPA: 100% |  |

Data are presented as the numbers of specimens. PPA, positive predictive agreement. NPA, negative predictive agreement.
